# Dysregulated Serum Lipid Metabolism Promotes the Occurrence and Development of Diabetic Retinopathy Associated With Upregulated Circulating Levels of VEGF-A, VEGF-D, and PlGF

**DOI:** 10.3389/fmed.2021.779413

**Published:** 2021-11-22

**Authors:** Xinyuan Zhang, Bingjie Qiu, Qiyun Wang, Sobha Sivaprasad, Yanhong Wang, Lin Zhao, Rui Xie, Lei Li, Wenting Kang

**Affiliations:** ^1^Beijing Tongren Eye Center, Beijing Institute of Ophthalmology, Tongren Hospital, Capital Medical University, Beijing, China; ^2^National Institute for Health Research (NIHR) Moorfield's Biomedical Research Center, Moorfield's Eye Hospital, London, United Kingdom; ^3^Department of Epidemiology and Biostatistics, School of Basic Medicine, Peking Union Medical College, Institute of Basic Medical Sciences, Chinese Academy of Medical Sciences, Beijing, China

**Keywords:** dyslipidemia, diabetes mellitus, diabetic retinopathy, lipid profile, serum cytokines, arteriosclerosis-associated plasma parameters

## Abstract

**Purpose:** This study aims to explore the correlations of arteriosclerosis-associated plasma indices with various severity levels of diabetic retinopathy (DR) and to test the hypothesis that elevated circulating level of known angiogenic cytokines induced by hyperglycemia is associated with dyslipidemia on DR.

**Methods:** This cross-sectional study consists of 131 patients with type 2 diabetes. The patients were categorized based on their DR status into those with no DR (diabetes mellitus, DM), non-proliferative diabetic retinopathy (NPDR), and proliferative diabetic retinopathy (PDR) groups. The biochemical profile including fasting glucose, glycated hemoglobin (HbA1c), lipid profile were estimated, plasma angiogenic cytokines (vascular endothelial growth factor, VEGF-A, -C, -D) and placental growth factor (PlGF) were analyzed by protein microarrays. The atherogenic plasma index (API) was defined as low-density lipoprotein cholesterol/high-density lipoprotein cholesterol (LDL-C/HDL-C); atherogenic index (AI) was calculated as (TC-(HDL-C))/HDL-C and atherogenic index of plasma (AIP) was defined as log (TG/HDL-C).

**Results:** No significant differences were detected in the duration of hypertension, age, and gender between the three groups. Serum TC and LDL-C, AI, and API in the NPDR group and PDR group were significantly higher than those in the DM group. The circulating level of PlGF, VEGF-A, and VEGF-C were significantly correlated with the severity of DR. VEGF-D is a risk factor independent of API (*Z* = −2.61, *P* = 0.009) and AI (*Z* = −2.40, *P* = 0.016). Multivariate logistic regression showed that AI and API are strong risk factors for the occurrence and severity of DR. Associated with AI and API, VEGF-D and PlGF contribute to DR: VEGF-D [AI: *P* = 0.038, odd ratio (OR) = 1.38; VEGF-D: *P* = 0.002, OR = 1.00. API: *P* = 0.027, OR = 1.56, VEGF-D:*P* = 0.002, OR = 1.00] and PlGF [AI: *P* = 0.021, OR = 1.43; VEGF-D: *P* = 0.004, OR = 1.50. API: *P* = 0.011, OR = 1.66; VEGF-D: *P* = 0.005, OR = 1.49].

**Conclusions:** Total cholesterol (TC) and LDL-C are risk factors for presence of any DR. Atherogenic index and API are novel and better predictive indicators for the occurrence and severity of DR in comparion with the traditional lipid profiles. Abnormal lipid metabolism are associated with the upregulation of circulating cytokines that are linked to the severity of DR.

## Introduction

Diabetic retinopathy (DR) is the most common ocular microvascular complication of diabetes mellitus (DM). Vision threatening diabetic retinopathy (VTDR) is a leading cause of blindness in the working-age (20–65 years) in both developed and developing countries (1). Duration of diabetes is an established non-modifiable risk factor for DR (1, 2). Hyperglycemia is the strongest modifiable risk factor but people with optimal glycemic control also develop DR. The fenofibrate intervention and event lowering in diabetes (FIELD) and the action to control cardiovascular risk in diabetes (ACCORD) studies demonstrated that abnormal lipid metabolism is associated with the onset and progression of DR but the underlying mechanisms are yet to be elucidated (3, 4).

Anti-vascular endothelial grow factor (VEGF) therapy has revolutionized the management of DR and diabetic macular edema (DME). Ranibizumab and bevacizumab are VEGF-A inhibitors while aflibercept blocks VEGF-A and placental growth factor (PlGF). Although these anti-VEGF agents are superior to macular laser and a considerable proportion of patients improve visual acuity, the results of Diabetic Retinopathy Collaboration Network (DRCR.net) Protocol I and T studies show that persistent macular edema was seen in approximately 51–73% at 12 weeks and 32–66% after 24 weeks of regular anti-VEGF injections (5–8).

The role of dyslipidemia in DR risk is now receiving increasing attention. However, unlike the well-established direct correlations between serum lipid dysregulated with macrovascular complication (9, 10), the correlation of traditional serum lipid parameters with DR is controversial (11, 12). In recent years, a large number of clinical and laboratory studies have confirmed that arteriosclerosis-associated plasma parameters, including atherogenic plasma index (API), low-density lipoprotein cholesterol/high-density lipoprotein cholesterol (LDL-C/HDL-C), atherogenic index (AI), total cholesterol (TC)-HDL-C/HDL-C, and atherogenic index of plasma (AIP), log (triglycerides(TG)/HDL-C) are critical indicators of cardiovascular and microvascular diseases (13, 14). These lipid ratios reflect two or three traditional lipid parameters, respectively, and could be better indicators to mirror the metabolic and clincial interactions between lipid fractions. Atherogenic plasma index is an independent risk factor for the occurrence of DM, and AIP has a predictive effect on the occurrence of macrovascular and microvascular diseases in the early stage of DM, but it is not known whether these indices are associated with the presence of DR (15, 16).

In the present study, we aimed to explore the association of arteriosclerosis-associated plasma indices with increasing severity of DR. We further investigated the correlations between these atherogenic indices and circulating levels of VEGF-A, VEGF-C, VEGF-D, and PlGF in patients with DM with and without DR to elucidate disease mechanisms that can be translated to novel therapeutic targets for DR.

## Subjects and Methods

### Participants

This prospective study followed the principles of the Declaration of Helsinki and was approved by the Ethics Committee of Beijing Tongren Hospital, Capital Medical University. All subjects signed an informed consent form before participation.

A total of 131 patients with type 2 DM, including 77 males and 54 females, aged 27–76 years old were recruited from the outpatient clinic of Beijing Tongren Hospital from April 2016 to September 2020. The participants were assigned to the DM group if they had no DR [32 patients, aged 37–75 years, median (IQR): 56 (48–65) years], non-proliferative diabetic retinopathy (NPDR) [56 patients, aged 29–76 years, 56 (51–61)] years, and proliferative diabetic retinopathy (PDR) [43 patients, aged 27–74 years, 55 (49–60) years].

### Inclusion and Exclusion Criteria

Participants with type 2 DM were included in the study. Type 2 DM was defined according to the 2020 American Diabetes Association (ADA) guidelines of DM (5) and 2017 A Position Statement of DR (6). Those with the ability to provide informed consent were included in the study. Exclusion criteria included people with type 2 DM with macular edema secondary to other retinal vascular diseases; co-existent other retinal diseases such as age-related macular degeneration, uveitis, and inherited retinal diseases; recent history of posterior segment or cataract surgery; ocular media opacity; and unable to tolerate examinations due to severe system diseases. Patients with history of lipid disorders or on lipid-lowering therapy were also excluded.

### Eye Examination

#### Routine Eye Examination

The participants underwent assessment of best-corrected visual acuity (BCVA), non-contact intraocular pressure (TX20 Automatic Non-contact Tonometer, Canon Co., Ltd., Tokyo, Japan), slit-lamp microscopic examination (SL-IE Slit Lamp Microscope, Topcon Co., Ltd., Tokyo, Japan), and fundus examination with mydriasis. Fundus photography (CR-1 non-mydriatic Fundus Camera, Canon Co., Ltd.) was done to capture at least two-field centered on optic disc and macula of both eyes. Ophthalmologists ascertained the DR status of the participants based on the International DR severity scale (6). Swept-source optical coherent tomography was applied (DRI OCT1 Atlantis scanner, Topcon Co., Ltd., Tokyo, Japan or Plex Elite 9000, Carl Zeiss Meditec, Inc., Oberkochen, German) for all the enrolled subjects. B-scan images were obstaind by a 9 × 9 mm scanning range mode. The DR status of the worse eye was recorded as an individual's DR grade. The participants were categorized into “PDR” group if they had retinal and/or optic disc neovascularization in at least one eye. Those with any other DR grade were categorized as NPDR group and participants with no DR in both eyes were assigned to the “DM” group.

Other data was also collected included age, gender, and duration of diabetes.

Blood biochemistry profile: Fasting biochemical examination was performed to detect the blood lipid profile of the participants. These include TC and LDL-C TGs, glycated hemoglobin (HbA1c), fasting blood glucose ect.

### Definition of Dyslipidemia

The definition was followed to the national cholesterol education program (NCEP) adult treatment panel-III (ATP-III) report in 2001 and the guidelines for the prevention and treatment of dyslipidemia in Chinese adults 2016. Dyslipidemia was defined as high cholesterol (TC) group (TC > 5.17 mmol/L), high TG group (TG > 1.70 mmol/L), or high low-density lipoprotein cholesterol (low-density lipoprotein cholesterol, LDL-C) group (LDL-C > 3.37 mmol/L).

### Determination of the Cutoff Value of AIP, API, and AI by Receiver Operating Characteristic Curve

Arteriosclerosis indices including API (LDL-C/HDL-C), AI [(TC-HDL-C)/HDL-C), and AIP (log(TG/HDL-C)] were calculated. Atherogenic plasma index, AI, and AIP with high sensitivity and specificity were selected as the cutoff values on receiver operating characteristic (ROC) curve. Patients with API > 2.24 (AUC: 0.746; sensitivity = 0.708, specificity = 0.517), AI > 2.91 (AUC, 0.723; sensitivity = 0.629; specificity = 0.724) or AIP > 0.01 (AUC 0.564; sensitivity = 0.607, specificity = 0.552) were assigned to high API, high AI, and high AIP groups, respectively.

### Determination of the Plasma Level of Cytokines

The Luminex technology (Luminex 200™ liquid chip detector, Millipore, Boston, Massachusetts, USA) was applied to detect the plasma level of cytokines, VEGF-A, VEGF-C, VEGF-D, and PlGF according to the manufacturer's instructions.

### Sample Size Calculation

Sample size was determined by using the Cochran's sample size formula and also reference the previous studies (12). Sample size was calculated at 95% confidence level with a margin of error of ±5%. To detect the difference (0.83) between means of expression level of angio-cytokines with a significance level of (alpha) 0.05. The minimum sample size was calculated to be 25 in each group. However, since there is variability in the circulating level of angio-cytokines/serum lipid profiles and highly variable parameters, the sample size was increased to above 30 in the present study.

### Statistical Analysis

Statistical analysis was performed using SPSS software (SPSS, Inc., 23.0, Chicago, IL, USA). Normality was assessed by Kolmogorov-Smirnov test and Shapiro-Wilk test. Variance homogeneity was tested by the Levene's test. Age of participant, duration of diabetes, fasting blood glucose, glycosylated hemoglobin, hypertension, TG, TC, and LDL-C were described by means ± standard deviation (mean ± SD) or median (interquartile range). The comparisons among groups were analyzed by one-way analysis of variance (ANOVA) or the Kruskal–Wallis test. The circulating levels of cytokines VEGF-A, VEGF-C, VEGF-D, and PlGF were reported as mean ± SD or median (interquartile range), comparisons between groups [DM, NPDR, and PDR groups, or DM and DR groups (combined NPDR with PDR as DR group)] was analyzed by independent sample *t*-test or Mann–Whitney U-test according to the data distribution (If the data were not normally distributed or variance homogeneity was not met). Bonferroni corrections was applied for comparison between the three groups. The effects of age, gender, the duration of diabetes, AI, API, AIP as well as their relationships with VEGF-A, VEGF-C, VEGF-D, and PlGF on DR patients were analyzed by multiple logistic regression analysis. *P* < 0.05 indicated statistical significance.

## Results

### Baseline Demographic and Clinical Characteristics

No statistically significant differences in age or gender were detected between the DM (with no DR), NPDR and PDR groups (*P*_*age*_ = 0.692; *P*_*gender*_ = 0.756). The average duration of DM was shorter in the DM group compared to that in the NPDR and the PDR group (12.59 ± 5.79 vs. 9.14 ± 6.76 years, *P*_*NPDRvs*.*DM*_ = 0.013; 12.86 ± 6.76 vs. 9.14 ± 6.76 years*, P*_*PDRvs*.*DM*_ = 0.021). There was no statistical significance of fasting blood glucose in the DM, NPDR, and PDR groups (*P* = 0.151). The difference between the NPDR and the DM groups [6.80 (6.18–7.83) vs. 7.75 (7.03–8.98) years, *P* = 0.018)], PDR and the DM groups [6.80 (6.18–7.83) vs. 7.80 (6.68–9.10) years, *P* = 0.017] of the glycosylated hemoglobin levels were statistically significant. No significant difference was detected in the duration of hypertension among the three groups (χ^2^ = 4.27, *P* = 0.118) (Table 1).

**Table 1:** Baseline demographic, clinical characteristics, biochemical parameters, and the circulating angiogenic cytokines of the enrolled subjects.

**Group**	**DM**	**NPDR**	**PDR**	***H*/*F*/*χ^2^***	***P*-value**
Number	32	56	43	–	–
Age (years)	55.5 (48.25–65.00)	56 (50.50–61.00)	55 (49.00–60.00)	0.74^a^	0.692
Female sex (%) (M/F)	43.75	37.5	44.17	0.56^b^	0.756
Duration of DM (years)	9.14 ± 6.76	12.59 ± 5.79	12.86 ± 6.76	3.82^c^	0.025^*^
Duration of HBP (years)	3.00 (0–10.00)	0.00 (0–5.00)	2.75 (0–10.00)	4.27^a^	0.118
Fasting blood glucose (mmol/L)	7.46 (5.81–8.72)	8.25 (6.38–9.67)	8.46 (6.42–11.20)	3.78^a^	0.151
HbA1c (%)	6.8 (6.18–7.83)	7.75 (7.03–8.98)	7.8 (6.68–9.10)	5.21^a^	0.074
Triacylglycerol (mmol/L)	1.32 (1.01–2.60)	1.2 (0.82–1.81)	1.4 (0.92–2.49)	2.33^a^	0.312
Cholesterol (mmol/L)	4.30 (3.91–5.03)	4.59 (3.74–5.68)	4.99 (4.32–5.89)	6.84^a^	0.033^*^
LDL-C (mmol/L)	2.50 (2.05–3.09)	2.80 (2.03–3.60)	3.06 (2.58–3.92)	9.03^a^	0.011^*^
HDL-C (mmol/L)	1.17 (1.00–1.40)	1.16 (0.98–1.48)	1.13 (0.98–1.43)	1.15^a^	0.562
API	2.19 ± 0.71	2.42 ± 0.91	2.82 ± 0.95	5.05^a^	0.008^*^
AI	2.75 ± 0.97	2.92 ± 1.17	3.44 ± 1.20	4.07^b^	0.019^*^
AIP	0.08 ± 0.39	0.004 ± 0.31	0.11 ± 0.30	1.41^b^	0.247
VEGF-A (pg/ml)	15.47 (11.40–25.92)	27.64 (20.62–35.32)	24.85 (18.59–33.54)	17.15^a^	<0.001^*^
VEGF-C (pg/ml)	52.61 (13.41–150.07)	87.76 (55.69–142.05)	99.82 (53.81–198.35)	4.87^a^	0.088
VEGF-D (pg/ml)	129.31 (47.14–218.77)	248.54 (150.45–428.89)	274.2 (172.90–401.96)	19.70^a^	<0.001^*^
PlGF (pg/ml)	1.41 (0.58–2.44)	2.69 (1.89–3.31)	2.41 (1.65–3.87)	16.38^a^	<0.001^*^

*DM, diabetes mellitus; NPDR, non-proliferative diabetic retinopathy; PDR, proliferative diabetic retinopathy; HbA1c, hemoglobin; LDL-C, low density lipoprotein cholesterol; HDL-C, high density lipoprotein cholesterol; VEGF, vascular endothelial growth; PlGF, placental growth factor; API/AI/AIP, atherogenic index of plasma; API, atherogenic plasma index, defined as LDL-C/HDL-C; AI, atherogenic index, was calculated as TC-(LDL-C)/HDL-C; AIP, atherogenic index of plasma, defined as log triglycerides TG/HDL-C; PIGF, placental growth factor; VEGF, vascular endothelial growth factor*.

* *Statistically significant: P ≤ 0.05. According to the type of data and the data distribution, one-way ANOVA analysis (a), Post-hoc LSD correction. b Kruskal–Wallis (a) were applied*.

### Correlations Between Lipid Profile and DR Severity

The level of TC in the three groups differed significantly [4.30 (3.91–5.03) vs. 4.59 (3.74–5.68) vs. 4.99 (4.32–5.89) mmol/L, *P* = 0.033], with the level of TC in the PDR group being higher than that in the DM group [4.99 (4.32–5.89) vs. 4.30 (3.91–5.03) mmol/L, *P* = 0.006]. In addition, LDL-C also showed statistical differences [2.50 (2.05–3.09) vs. 2.80 (2.03–3.60) vs. 3.06 (2.58–3.92) mmol/L, *P* = 0.011], with the level in the PDR group being higher than that in the DM group [3.06 (2.58–3.92) vs. 2.80 (2.03–3.60) mmol/L, *P* = 0.002]. No statistically significant difference was detected in the three groups with respect to TG and HDL (*P*_*TG*_ = 0.312; *P*_*HDL*−*C*_ = 0.562) (Table 1).

### Associations Between Atherogenic Indices and DR

The API in the three DR groups showed a statistically significant difference (2.19 ± 0.71 vs. 2.42 ± 0.91 vs. 2.82 ± 0.95, *P* = 0.008), with the value of the PDR group higher than that of the DM group (*P* = 0.003). The difference of AI in the three groups also differed significantly (2.75 ± 0.97 vs. 2.92 ± 1.17 vs. 3.44 ± 1.20, *P* = 0.019), with the value in the PDR group significantly higher than that in the NPDR and DM group (*P*_*PDRvs*.*NPDR*_ = 0.024, *P*_*PDRvs*.*DM*_ = 0.010). There was no difference of the AIP-value between the three groups (*P* = 0.247) (Table 1).

### Correlations Between the Circulating Level of Angiogenic Cytokines and DR Severity

The plasma level of VEGF-A differed significantly [15.47 (11.40–25.92) vs. 27.64 (20.62–35.32) vs. 24.85 (18.59–33.54) pg/ml, *P* < 0.001], with the level of VEGF-A in NPDR and PDR groups significantly higher than that in the DM group (*P*_*NPDRvs*.*DM*_ = 0.002, *P*_*PDRvs*.*DM*_ = 0.001). There was also a significant difference in VEGF-D between the three groups (*P* < 0.001), with higher level in NPDR and PDR groups than that in DM group (*P*_*NPDRvs*.*DM*_ < 0.001, *P*_*PDRvs*.*DM*_ < 0.001). The level of PlGF in the three groups differed significantly [1.41 (0.58–2.44) vs. 2.69 (1.89–3.31) vs. 2.41 (1.65–3.87) pg/ml, *P* < 0.001], with the level of PlGF in the NPDR and PDR groups significantly higher than that in the DM group [2.69 (1.89–3.31) vs. 1.41 (0.58–2.44) pg/ml, *P*_*NPDRvs*.*DM*_ = 0.012, 2.41 (1.65–3.87) vs. 1.41 (0.58–2.44) pg/ml, *P*_*PDRvs*.*DM*_ < 0.001]. No statistical significance was detected in the three groups with the circulating level of VEGF-C (*P* = 0.088) (Table 1).

### Associations Between Atherogenic Indices and the Circulating Level of Angiogenic Cytokines

There was a significant difference in the plasma level of VEGF-D between the normal and abnormal API group [154.10 (71.78–274.36) vs. 255.58 (147.91–400.44) pg/ml, *P* = 0.009]. Similarity, differed expression level of VEGF-D was found between the normal AI and abnormal group [179.77 (72.09–280.18) vs. 255.59 (147.91–400.45) pg/ml, *P* = 0.016]. There is no significant difference in the expression level of VEGF-A, VEGF-C, and PlGF in between the normal API, AI, and AIP groups. No significant difference was found in the expression level of VEGF-D between the normal AIP and abnormal AIP groups (Table 2).

**Table 2:** Comparison of the circulating level of VEGF-A, VEGF-C, VEGF-D, and PlGF in patients with abnormal and normal arteriosclerosis-associated plasma indices (API, AI, AIP).

	**Cytokines**	**VEGF-A (pg/ml)**	**VEGF-C (pg/ml)**	**VEGF-D (pg/ml)**	**PIGF (pg/ml)**
**API**					
	Normal API group	22.45 (14.59–32.91)	80.58 (36.06–127.49)	154.1 (71.78–274.36)	2.13 ± 1.32
	Abnormal API group	24.85 (18.24–34.29)	90.86 (53.81–182.03)	255.58 (147.91–400.44)	2.54 ± 1.28
	*t/z*	−1.25^c^	−1.54^c^	−2.61^c^	−1.63^b^
	*P-value*	0.211	0.124	0.009^*^	0.106
**AI**					
	Normal AI group	22.77 (15.35–31.86)	80.58 (37.28–127.49)	179.77 (72.09–280.18)	2.16 ± 1.26
	Abnormal AI group	26.8 (18.24–35.72)	101.57 (53.81–187.19)	255.59 (147.91–400.45)	2.59 ± 1.32
	*t/z*	−1.70^c^	−1.85^c^	−2.40^c^	−1.76^b^
	*P-value*	0.09	0.064	0.016^*^	0.081
**AIP**					
	Normal AIP group	23.08 (17.63–32.30)	82.65 (38.17–145.62)	192.47 (83.52–286.23)	2.25 ± 1.28
	Abnormal AIP group	24.85 (17.63–34.53)	99.82 (51.41–175.89)	252.01 (137.00–389.96)	2.50 ± 1.33
	*t/z*	−0.94^c^	−1.15^c^	−1.60^c^	−1.01^b^
	*P-value*	0.346	0.249	0.111	0.316

* *Statistically significant: P ≤ 0.05. According to the type of data and the data distribution*,

a *Kruskal-Wallis test*,

b *independent-sample t-test, and*

c *Mann-Whitney U-test were applied. VEGF, vascular endothelial growth; PlGF, placental growth factor; DM, diabetes mellitus; NPDR, non-proliferative diabetic retinopathy; PDR, proliferative diabetic retinopathy; API, atherogenic plasma index (LDL-C/HDL-C); AI, atherogenic index: (TC-(LDL-C))/HDL-C; AIP, atherogenic index of plasma: log (TG/HDL-C)*.

### Associations Between VEGF-A, -B, -C, -D, PGF in All Patients With Abnormal and Normal TC, TG, LDL-C, and HDL-C

The level of VEGF-A, VEGF-C, VEGF-D, PlGF is significantly higher in the abnormal TC group in comparison with the normal TC group (*P*_*VEGF*−*A*_ = 0.008; *P*_*VEGF*−*C*__=_ 0.001*, P*_*VEGF*−*D*_ = 0.035; *P*_*PlGF*_ = 0.004). Similarity, in the abnormal LDL-C group, the level of VEGF-A, VEGF-C, VEGF-D, and PlGF is significantly higher than that in the normal LDL-C group (*P*_*VEGF*−*A*_ = 0.032; *P*_*VEGF*−*C*_ = 0.006; *P*_*VEGF*−*D*_ = 0.018, P_PlGF_ = 0.006). No statistical difference was found between the circulating level of VEGF-A, -C, -D, and PlGF in the abnormal TG, HDL-C groups, compared with that in the normal TG and HDL-C groups, respectively, indicating the circulating level of the angiogenic cytokines is correlated with the abnormal serum level of TC and LDL-C (Table 3).

**Table 3:** Comparison of the circulating level of VEGF-A, VEGF-C, VEGF-D, and PlGF in all patients with abnormal and normal TC, LDL, TG, and HDL.

		**VEGF-A**	**VEGF-C**	**VEGF-D**	**PlGF**
		**(pg/ml)**	**(pg/ml)**	**(pg/ml)**	**(pg/ml)**
**TC:**					
	Normal TC group	22.45 (17.09–32.30)	77.16 (43.40–125.32)	201.47 (122.13–335.91)	2.14 (0.99–3.10)
	Abnormal TC group	27.64 (19.86–37.45)	122.65 (61.12–219.99)	266.55 (142.79–427.94)	2.84 (1.78–3.66)
	*z*	−2.66^b^	−3.31^b^	−2.11^b^	−2.87^b^
	*P-value*	0.008^*^	0.001^*^	0.035^*^	0.004^*^
**LDL-C:**					
	Normal LDL-C group	22.77 (17.41–32.30)	78.50 (42.61–128.59)	196.32 (102.48–335.91)	2.14 (1.00–3.13)
	Abnormal LDL-C group	27.64 (19.33–37.06)	118.52 (55.69–212.44)	266.55 (178.27–433.74)	2.84 (1.69–3.66)
	*z*	−2.14^b^	−2.77^b^	−2.36^b^	−2.74^b^
	*P-value*	0.032^*^	0.006^*^	0.018^*^	0.006^*^
**TG:**					
	Normal TG group	24.85 (18.59–34.20)	82.65 (46.90–140.71)	245.08 (128.21–395.90)	2.47 (1.34–3.28)
	Abnormal TG group	24.49 (16.68–32.01)	115.51 (48.03–207.07)	209.77 (121.46–361.79)	1.85 (1.31–3.51)
	*z*	−0.84^b^	−1.48^b^	−0.51^b^	−0.74^b^
	*P-value*	0.400	0.140	0.610	0.458
**HDL-C:**					
	Normal HDL-C group	24.85 (17.76–33.56)	99.82 (47.23–186.00)	227.54 (110.30–405.02)	2.41 ± 1.34
	Abnormal HDL-C group	24.86 (18.06–33.30)	81.95 (46.60–117.01)	233.87 (136.74–363.15)	2.33 ± 1.17
	*t/z*	−0.29^b^	−0.99^b^	−0.10^b^	−0.37^a^
	*P-value*	0.772	0.321	0.921	0.712

*VEGF, vascular endothelial growth; PlGF, placental growth factor; TC, cholesterol; TG, triglycerides; LDL-C, low-density lipoprotein cholesterol; HDL-C, high density lipoprotein cholesterol*.

* *Statistically significant: P ≤ 0.05. According to the type of data and the data distribution*,

a *independent-sample t-test, and*

b *Mann-Whitney U-test were applied*.

### Multiple Ordinal Logistic Regression Models Show the Correlations of AI, AIP, API, and Circulating Levels of VEGF-A, VEGF-C, VEGF-D, PlGF in Normal, DM, or DR Groups

Table 4 shows the correlations of the plasma level of VEGF-A, VEGF-C, VEGF-D, and PlGF with atherogenic indices on the occurrence and severity of DR using multiple ordinal logistic regression models. When DM, NPDR, and PDR were considered as independent variables, after controlling the duration of diabetes and hypertension, the serum level of hemoglobins and fasting glucose, AI and API are strong risk factors for the occurrence and severity of DR. Atherogenic index is associatd with VEGF-D [AI: *P* = 0.038, OR = 1.38 (1.02–1.86); VEGF-D: *P* = 0.002, OR = 1.00 (1.00–1.01)] and PlGF [AI: *P* = 0.021, OR = 1.43 (1.06–1.92); PlGF: *P* = 0.004, OR = 1.50 (1.14–1.98)] contributed to DR (Table 4A). Atherogenic plasma index is associated with VEGF-D (Table 4B): [API: *P* = 0.027, OR = 1.56 (1.05–2.30); VEGF-D:*P* = 0.002, OR = 1.00 (1.00–1.01)] and PlGF [API: *P* = 0.011, OR = 1.66 (1.12–2.47); PlGF: *P* = 0.005, OR = 1.49 (1.13–1.96)] (Table 4B). The correlations etween AIP and VEGF-C, VEGF-D, VEGF-A, PlGF were summarized in Table 4C.

**Table 4:** Multiple ordinal logistic regression models showing that AI and API contributed to the occurrence and severity of DR asscociated with elevated circulating level of VEGF-D and PIGF.

		**Variable**	**Estimate**	**StdErr**	***P-*value**	**OR**
**(A)**						
	Model 1	AI	0.39	0.15	0.010^*^	1.48 (1.10–1.99)
		VEGF-A	0.02	0.01	0.098	1.02 (1.00–1.04)
	Model 2	AI	0.35	0.16	0.027^*^	1.41 (1.04–1.92)
		VEGF-C	0.00	0.00	0.093	1.00 (1.00–1.01)
	Model 3	AI	0.32	0.15	0.038^*^	1.38 (1.02–1.86)
		VEGF-D	0.00	0.00	0.002^*^	1.00 (1.00–1.01)
	Model 4	AI	0.35	0.15	0.021^*^	1.43 (1.06–1.92)
		PlGF	0.41	0.14	0.004^*^	1.50 (1.14–1.98)
**(B)**						
	Model 1	API	0.56	0.20	0.004^*^	1.74 (1.19–2.59)
		VEGF-A	0.02	0.01	0.093	1.02 (1.00–1.04)
	Model 2	API	0.50	0.20	0.013^*^	1.65 (1.11–2.45)
		VEGF-C	0.00	0.00	0.105	1.00 (1.00–1.01)
	Model 3	API	0.44	0.20	0.027^*^	1.56 (1.05–2.30)
		VEGF-D	0.00	0.00	0.002^*^	1.00 (1.00–1.01)
	Model 4	API	0.50	0.20	0.011^*^	1.66 (1.12–2.45)
		PlGF	0.40	0.14	0.005^*^	1.49 (1.13–1.96)
**(C)**						
	Model 1	AIP	0.39	0.50	0.444	1.47 (0.55–3.95)
		VEGF-A	0.02	0.01	0.084	1.02 (1.00–1.04)
	Model 2	AIP	0.14	0.52	0.783	1.15 (0.42–3.20)
		VEGF-C	0.00	0.00	0.037	1.00 (1.00–1.01)
	Model 3	AIP	0.34	0.51	0.507	1.40 (0.52–3.82)
		VEGF-D	0.00	0.00	0.001^*^	1.00 (1.00–1.01)
	Model 4	AIP	0.27	0.51	0.593	1.31 (0.49–3.55)
		PlGF	0.44	0.14	0.002^*^	1.55 (1.18–2.04)

*API, atherogenic plasma index (LDL-C/HDL-C); AI, atherogenic index (TC-(LDL-C))/HDL-C; DM, diabetes mellitus; NPDR, non-proliferative diabetic retinopathy; PDR, proliferative diabetic retinopathy; PIGF, placental growth factor; VEGF-D, vascular endothelial growth factor D; HBP, hypertension; HbA1c, hemoglobin A1c. When DM, NPDR, and PDR were considered as the independent variable, after controlling the age, sex, duration of diabetes and hypertention, fast glucose, and hemoglobin, the multiple ordinal regression models show that AI and API contributed to the occurrence and serverity of DR associated with elevated plasma level of VEGF-D and PIGF*.

* *P ≤ 0.05 is considered statistical significance*.

### The Arteriosclerosis-Associated Plasma Indices AI and API Are More Reliable DR Predictors Than the Traditional Lipid Parameters LDL-C, TC TG, and HDL-C

The above analysis has proved that API and AI strongly associated with DR severity. A greater area under the ROC (AUC) means a more useful test of the statistical model. The AUC of AI (AUC:0.72; 95% CI: 0.62–0.82) and API (AUC: 0.75;95%CI: 0.65–0.84) is much higher than TC (AUC:0.68; 95%CI: 0.58–0.78), TG (AUC: 0.54; 95% CI 0.41–0.67) LDL-C (AUC:0.72; 95% CI: 0.62–0.82), HDL-C (AUC:0.57, 95% CI: 0.45–0.69), indicating that both API and AI have higher capacity to differential DR.

## Discussion

In this study, we found that serum TC, LDL-C, AI, and API in the NPDR and PDR groups were significantly higher than those in the DM group. The circulating level of PlGF, VEGF-A, and C were significantly correlated with the severity of DR. Multivariate logistic regression showed that diabetes duration, AI and API are strong risk factors for DR. Furthermore, AI and API have synergic effects with VEGF-D and PlGF with various severity levels of DR, indicating AI and API are novel predictors of DR. This study, for the first time, clarified and elucidated the pathological mechanism of dyslipidemia in the pathogenesis of DR.

It has been reported that the prevalence of DR is 35% globally by a meta-analysis (17). The duration of DM is a key risk factor for DR. In addition, systemic factors such as hyperglycemia, hypertension and hyperlipidemia contribute and accelerate the progression and deterioration of DR. Several studies have indicated that a high level of TC and LDL-C promotes the occurrence and progression of retinopathy and coronary heart disease in DM (18–20). The exact mechanism(s) underlying hyperlipidemia-mediated DR has not yet been elucidated. The current study provided the first evidence that abnormal lipid metabolism promotes the occurrence and development of DR by upregulating the expression of VEGF-A, VEGF-C, VEGF-D, and PlGF in the circulating of patients with DR.

### Abnormal Lipid Metabolism Is Closely Correlated to the Occurrence and Progression of DR

The Wisconsin Epidemiologic Study of DR first identified that a long-term increase in the serum level of TC might be a risk factor for hard exudates in DR in 1991 (21), FIELD and ACCORD study further confirmed that lipid-lowing therapy with fenofibrate significantly reduce the progression of DR and the need for laser treatment (3, 22), indicating that intensive lipid control is correlated with improved clinical prognosis. Statins are the most effective drug to reduce TC and LDL-C. However, the role of statins is more profound in cardiovascular disease than DR (3, 22–24).

Our study found that the levels of TC and LDL-C in the NPDR and PDR groups were significantly higher than those in the DM group, which are consistent with the results of previous studies on type 1 DM (25). In addition, the serum levels of TC and LDL-C in PDR patients were significantly increased compared to those patients with NPDR and DM, suggesting that the increased levels of TC and LDL-C in serum accelerate the progression of DR.

### AI and API Are Independent Risk Factors for DR

In this study, the lipid metabolism indices were used to explore the relationship between dysregulated lipid metabolism and the expression level of circulating cytokines and DR severity. In routine clinical practice, LDL-C is calculated (by the Friedewald formula) than measured directly. Several studies have found that the estimation of LDL-C has significant limitations, especially in individuals with hypertriglyceridemia with DM (it may contain abnormal composition of the TG-rich lipoproteins which is not considered in the Friedewald formula) (14, 26–28).

Non-HDL-C represents the cholesterol components carried by atherogenic lipoproteins such as LDL, very low density lipoprotein, and intermediate density lipoprotein and has been recommended as the primary lipid-lowing target by the 2019 American College of Cardiology (ACC) and American Heart Association (AHA) guidelines on the management of blood cholesterol (29).

It has been confirmed by studies in cardiovascular diseases that AI and API reflect two or three traditional parameters, which are simple and economic and can be used as biomarkers. Atherogenic index and API have several key advantages than the traditional lipid parameters: they have explicit pathophysiologic link to the development of atherosclerosis, robust from a laboratory measurement standpoint etc. (14) API, AI, and AIP have been applied for assessing plasma lipid homeostasis and are also implicated in the pathogenesis of macrovascular complications, especially in the early stage of the disease when the unaltered concentration of various lipids is increased (30). Atherogenic plasma index is a sensitive predictor of asymptomatic type 2 DM patients with coronary atherosclerosis and the incidence of microvascular complications and peripheral neuropathy in DM patients increased significantly with the increased AIP (15, 31). Since this is the first study to apply the indices in eye study, we used the ROC curve, the objective method to obtain the cut off value, and also referred to the value of the lipid metabolism in cardiovascular and atherosclerosis studies [API: 0.16–2.24 (32), AI: 3.21–3.37 (33), AIP: 0.6–0.21 (34, 35)].

In this study, we found that the AUC of AI (TC-(HDL-C)/HDL-C) (a) and API (LDL-C/HDL-C) is significantly higher than TC, TG, LDL-C, and HDL-C, indicating these simpler combined parameters have higher diagnostic capacity than the traditional parameters for DR. Furthermore, logistic regression analysis showed that AI and API are independent risk factors for DR, and provide evidence for a link between lipid metabolism and DR.

### Dysregulated Circulating Lipid Metabolism Promotes the Occurrence and Development of DR by Upregulating VEGF-A, VEGF-C, VEGF-D, and PlGF

VEGF-A is not only the most important vascular leakage inducer but also a key angiogenesis factor and a target of anti-VEGF therapy in ocular conditions (36, 37). However, macular oedema does not resolve completely in a number of patients with DR suggesting that there are other influencers in the pathogenesis of DR. Our study shows that other family members of VEGF are also raised in DR, indicating the need for novel molecular targets. Both VEGF-C and VEGF-D are secreted glycoproteins that exhibit structural homology but have differential receptor binding (Flk-1 and Flt-3, respectively) and regulatory mechanism is a vital mediator to angiogenesis in ischemic heart disease in type 2 DM (38). These cytokines have been demonstrated as independent predictors for suspected coronary artery disease and are associated with all-cause mortality (39). In a previous study, VEGF-A-FLK-1 signaling has been shown as a primary mediator of endothelial cell mitogenesis, survival, and microvascular permeability (40). Together with VEGF-A, VEGF-C, and VEGF-D promote blood vessel development (angiogenesis) by binding and activating VEGF-R-2 and VEGF-R-3. VEGF-C is also a potent inducer of vascular permeability and angiogenesis in AMD. The ongoing phase III trial is investigating a soluble form of VEGF-R-3, which comprises the extracellular domains 1–3 of human VEGF-R3. In addition, the Fc fragment of human IgG1 suppresses the binding of VEGF-C and -D to VEGF-R 2 and 3, respectively (ClinicalTrials.gov Identifier: NCT02543229). The positive outcome from the phase II trial has shown that the combined intravitreal administration of OPT-302 and ranibizumab is better than ranibizumab alone for the patient's vision prognosis. Furthermore, PlGF is deemed to play a crucial role in both experimental animal models and retinal vascularization (41, 42). A high expression level of PlGF has also been detected in vitreous and PDR membrane in patients with PDR (43–46).

In the present study, based on a significant body of evidence in preclinical and clinical studies that support the pathological function of VEGF-A, -C, -D, and PlGF, a protein chip-Luminex technology was applied to quantitatively detect the circulating expression levels in patients with abnormal lipid metabolism group and normal group, respectively. We found that the circulating level of VEGF-D significantly contributed to the occurrence and servierty of DR associated with AI and API, confirmed the previous finding that VEGF-D is also an important regulator of genes related to lipid metabolism and inflammation (47–50). Our results also provide evidence for the first time that besides diabetes duration, AI, API, VEGF-D, and PlGF are strong risk factors for the occurrence and progression of DR. Atherogenic index and API have synergic effects with VEGF-D and PlGF. Placental growth factor may release VEGF-A by binding to VEGF-R-1 to activate VEGF-R-2, thereby indirectly stimulating angiogenesis, or combined with VEGF-R-1 to promote the proliferation of endothelial cells and angiogenesis (44). Animal experiments also have shown that PlGF promotes the infiltration of macrophages in early atherosclerotic lesions in apolipoprotein E deficiency (Apo E^−/−^) mice and rabbits with high TC. In this study, by logistic regression analysis, after controlling the age, sex, duration of diabetes and VEGF-A, -C, -D, the serum level of TC and LDL was found to concomitant with higher level expression of PlGF in either DM or DR group, HDL was found to be negatively with PlGF expression. Thus, it could be speculated that high non-HDL-C promotes the development of DR by upregulating the expression of PlGF.

Presently, the specific regulatory mechanism of abnormal lipid metabolism involved in the progression of DR is yet to be clarified. Nevertheless, the present study has some limitations. Due to the small sample size, confounding factors could not be eliminated in the analysis process, and various measurement methods and correction factors might also affect the results of this study. Well-matched and large-scale prospective clinical trials and cohort studies are warranted to validate the conclusions in the study.

In summary, the ratios of LDL-C/HDL-C and TC-LDL-C/HDL-C are useful and simple indicators of DR. The present study further found that dysregulation of lipid metabolism promotes the development of DR and are associated with upregulation of circulating VEGF-A, VEGF-C, VEGF-D, and PlGF. The current results may partly explain why the response to anti-VEGF varies between individuals. Strengthening the management of blood lipids in patients with DM may help to reduce the levels of VEGF and PlGF and resultant oedema. Therefore, patients with DM should focus on the management of blood lipids in addition to their glycemic status. Further understanding the correlation between the arteriosclerosis-associated plasma indices and circulating levels of angiogenic cytokines can be translated to novel therapeutic targets for DR.

## Data Availability Statement

The original contributions presented in the study are included in the article/supplementary materials, further inquiries can be directed to the corresponding author.

## Ethics Statement

The studies involving human participants were reviewed and approved by Beijing Tongren Hospital. The patients/participants provided their written informed consent to participate in this study.

## Author Contributions

XZ contributed to conception and design of the study, perform the statistical analysis, revise and draft the manuscript. BQ and QW organized the database, performed the experiments, performed the statistical analysis and drafted the manuscript. SS provided comments and revised the manuscript. LZ, RX, and WK helped to enroll patients and were involved in the experiments. YW and LL performed the statistical analysis. All authors contributed to manuscript revision, read, and approved the submitted version.

## Funding

This work was supported by the National Natural Science Foundation of China (Grants 81570850, 81170859, and 82070988) and the Ministry of Science and Technology Foundation of China (Grant 2016YFC1305604).

## Conflict of Interest

The authors declare that the research was conducted in the absence of any commercial or financial relationships that could be construed as a potential conflict of interest.

## Publisher's Note

All claims expressed in this article are solely those of the authors and do not necessarily represent those of their affiliated organizations, or those of the publisher, the editors and the reviewers. Any product that may be evaluated in this article, or claim that may be made by its manufacturer, is not guaranteed or endorsed by the publisher.

## Abbreviations

ADA: American Diabetes Association
ACCORD study: action to control cardiovascular risk in diabetes study
AI: atherogenic index
AIP: atherogenic index of plasma
API: atherogenic plasma index
BCVA: best corrected visual acuity
DM: diabetes mellitus
DME: diabetic macular edema
DR: diabetic retinopathy
FIELD study: fenofibrate intervention and event lowering in diabetes study
HDL: high density lipoprotein
IQR: interquartile range
LDL: low density lipoprotein
NPDR: non-proliferative diabetic retinopathy
NCEP: national cholesterol education program
PDR: proliferative diabetic retinopathy
PlGF: placental growth factor
TC: total cholesterol
TG: triglycerides
VEGF: vascular endothelial growth factor
VTDR: vision threatening diabetic retinopathy.
